# Switch to Lisdexamfetamine in the Treatment of Attention-Deficit Disorder at a Psychiatric Outpatient Clinic for School-Aged Children: A Danish Cohort Study

**DOI:** 10.1089/cap.2023.0077

**Published:** 2024-04-11

**Authors:** Nanna Roed Søndergaard, Karen Busk Nørøxe, Anders Helles Carlsen, Stine Helene Randing, Pernille Warrer, Per Hove Thomsen, Loa Clausen

**Affiliations:** ^1^ Department of Child and Adolescent Psychiatry, Aarhus University Hospital, Psychiatry, Aarhus, Denmark.; ^2^ Department of Patient Safety, Zealand Pharma, Søborg, Denmark.; ^3^ Department of Clinical Medicine, Aarhus University, Aarhus, Denmark.

**Keywords:** lisdexamfetamine, child and adolescent psychiatry, attention-deficit/hyperactivity disorder, adverse effects, treatment

## Abstract

**Objectives:**

This study aimed to examine switch from first-line methylphenidate (MPH) to lisdexamfetamine (LDX) in school-aged children with attention-deficit/hyperactivity disorder (ADHD).

**Methods:**

This is a retrospective observational study based on systematic review of patient records of all children (7–13 years) diagnosed with ADHD and referred to a Danish specialized outpatient clinic. The study included 394 children switching from MPH to LDX as either second-line or third-line treatment (atomoxetine [ATX] as second-line treatment) during the study period from April 1, 2013, to November 5, 2019.

**Results:**

One in five children switched from MPH to LDX at some point during the study period. The most frequent reasons for switching to LDX were adverse effects (AEs; 70.0% for MPH, 68.3% for ATX) and lack of efficiency (52.0% for MPH, 72.7% for ATX). Top five AEs of LDX were decreased appetite (62.4%), insomnia (28.7%), irritability/aggression (26.1%), weight decrease (21.1%), and mood swings (13.9%). MPH and LDX had similar AE profiles, yet most AEs were less frequent after switching to LDX. At the end of the study period, the majority were prescribed LDX as second-line rather than third-line treatment (86.1% in 2019). However, the likelihood of LDX as second-line treatment decreased with the number of psychiatric comorbidities, ADHD symptom severity as assessed by parents, and if AEs were a reason for MPH discontinuation. Among children observed for at least 1 year after initiation of LDX, 41.3% continued LDX treatment for a year or longer. LDX continuation was less likely if AEs were a reason for MPH discontinuation. Similarly to MPH and ATX, the most frequent reasons for LDX discontinuation were AEs (74.4%) and lack of efficiency (34.7%).

**Implications:**

The findings support LDX as an important option in the personalized treatment of children with ADHD and may support prescribers in the clinical decision-making on switching medication.

## Background

Attention-deficit/hyperactivity disorder (ADHD) is a common neurodevelopmental disorder characterized by inattention, which is often combined with impulsivity and hyperactivity. In this article, the term ADHD refers to both inattentive and combined subtypes. ADHD most commonly manifests in childhood and lasts into adulthood and may interfere with social, emotional, and academic life, resulting in major functional impairment (Faraone et al., 2015). Recent estimates suggest that about 3%–7% of school-aged children are affected (Polanczyk et al., 2015; Polanczyk et al., 2007; Sayal et al., 2018; Thomas et al., 2015).

Pharmacological treatment plays a significant role in the management of moderate to severe ADHD in children and adolescents (Catala-Lopez et al., 2017), and clinical guidelines for pharmacological treatment of ADHD have changed over time as new drugs have been licensed. In most Western countries, current pharmacological treatment options licensed for treatment of children ≥6 years include psychostimulants (e.g., methylphenidate [MPH], amphetamines, and lisdexamfetamine [LDX]) and nonpsychostimulant medications (e.g., atomoxetine [ATX] and guanfacine) (Danish Health Authority, 2021a; Danish Health Authority, 2021b; National Institute for Health and Care Excellence, 2018; Pliszka and AACAP Work Group on Quality Issues, 2007). ATX and LDX were marketed in Denmark in 2006 and 2013, respectively.

The current guidelines for the pharmacological treatment of ADHD in children and adolescents lack consistency in their recommendations (Bolea-Alamanac et al., 2014; Canadian ADHD Resource Alliance, 2018; National Institute for Health and Care Excellence, 2018; Pliszka and AACAP Work Group on Quality Issues, 2007; Subcommittee on Attention-Deficit/Hyperactivity Disorder et al., 2011). However, most guidelines recommend MPH as first-line treatment (National Institute for Health and Care Excellence, 2018; The Danish Council for the Use of Expensive Hospital Medicines, 2016). This recommendation is supported by a large network meta-analysis (Cortese et al., 2018). The American Academy of Child and Adolescent Psychiatry (AACAP) generally recommends treatment with any drug approved by the U.S. Food and Drug Administration (FDA) for the treatment of ADHD in children based on an individualized choice (Pliszka and AACAP Work Group on Quality Issues, 2007).

Approximately 10%–30% of children and adolescents with ADHD may experience inadequate response or significant adverse effects (AEs) of MPH, why switching to alternative medication is sometimes required (Clavenna and Bonati, 2014; Dalsgaard et al., 2014; Warrer et al., 2016; Wigal, 2009). The NICE guideline recommends switching to LDX as second-line treatment, whereas LDX and ATX are recommended as equal second-line treatment options in the Danish national guideline (Danish Health Authority, 2021b; National Institute for Health and Care Excellence, 2018). The AACAP recommends switching to ATX if the patient experiences severe AEs from stimulants, but otherwise the choice of treatment rests solely with the family and the clinician (Pliszka and AACAP Work Group on Quality Issues, 2007).

Overall, clinical decision-making on switch of medication may be influenced by patient characteristics and preferences, medication profile (e.g., rapidity of onset, duration of effect, and AEs), cost, and the treating physician's experience with prescribing optional medications (Pliszka and AACAP Work Group on Quality Issues, 2007; Steingard et al., 2019).

Little is known about the frequency or reasons for switching from first-line MPH in the treatment of ADHD in children. In a Danish study examining 55 children and adolescents who switched from first-line MPH to second-line ATX, the most common reasons for switching were AEs (78%), followed by a wish for more optimal day coverage (24%) and lack of efficacy (16%) (Warrer et al., 2016). To the best of our knowledge, no previous studies have examined the reasons for switching to LDX. More knowledge is needed to inform clinical decision-making on switching to LDX.

Based on systematic review of patient records at a Danish child and adolescent psychiatric hospital, we aimed to examine switch in therapy from first-line MPH to second- or third-line in children with ADHD, including the frequency and reasons for making the switch, AEs, and LDX treatment continuation.

## Methods

### Design and setting

This retrospective observational study was based on data from medical records of children aged 7–13 years referred to and treated for ADHD at a specialized outpatient clinic at the Department of Child and Adolescent Psychiatry, Aarhus University Hospital. The hospital is located in the Central Denmark Region with a catchment area that comprises ≈1.3 million inhabitants. Patients are referred to the outpatient clinic from municipality services, general practitioners, and to a lesser extent from other hospital units.

### Study population and data collection

We included all children who were referred to the outpatient clinic during the study period from April 1, 2013, to November 5, 2019, diagnosed with ADHD and prescribed LDX at some point during the study period. All patients were evaluated according to the National Clinical Guideline for Assessment and Treatment of ADHD in Children and Young People (Danish Health Authority, 2021b). ADHD was diagnosed by child and adolescent psychiatrists at an interdisciplinary diagnostic conference. An ADHD-rating scale (ADHD-rs) filled out by teachers and parents was used to assess symptom severity (DuPaul et al., 1998). ADHD was diagnosed according to the International Classification of Diseases, 10th revision (ICD-10), including: F90.0 Disturbance of activity and attention; F90.1 Hyperkinetic conduct disorder; F90.8 Other hyperkinetic disorders; F90.9 Hyperkinetic disorder, unspecified; and F98.8c Attention-deficit disorder without hyperactivity.

Patients have routine follow-up visits at the outpatient clinic 2–3 months after initiation of pharmacological treatment and then approximately every 6 months. The ADHD-rs and questionnaire about the most common and severe AEs are routinely sent to parents and teachers before initiation of treatment and before each follow-up visit.

**FIG. 1.**
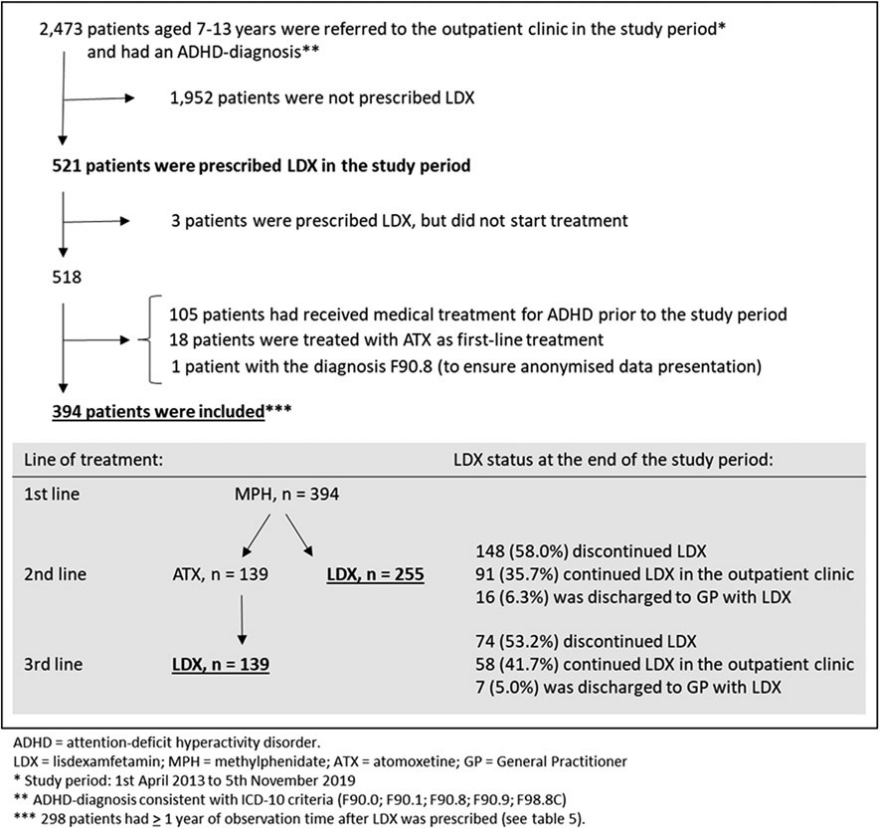
Flowchart of patients included in the study, and an overview of line of treatment and LDX treatment status at the end of the study period.

We excluded patients who were prescribed LDX but never redeemed their prescription (*n* = 3) and patients treated with ADHD medication before the study period (*n* = 105), as we lacked access to information on AEs and reasons for discontinuation of such prior treatment. In addition, we excluded patients with ATX as first-line treatment (*n* = 18). Finally, one patient with the diagnosis F90.8 was excluded to ensure anonymized data presentation. A total of 394 patients were eligible for inclusion (Fig. 1).

Eligible patients were identified in the electronic patient record (EPR) system via the business intelligence (BI) platform, which contains information on age, gender, referral date, diagnosis (including psychiatric comorbidities), and pharmacological data on all referred patients [Central Denmark Region (a), n.d.]. Because the pharmacological data in the BI platform were incomplete until September 1, 2016, a manual review of patient records was performed for patients referred before this date.

Data on AEs, reasons for treatment discontinuation, and ADHD symptom severity (assessed by ADHD-rs) before treatment initiation were collected by a systematic review of the EPR clinical notes (free-text form), and data on the initiation and discontinuation of medication were obtained from the EPR medicine module. Data were entered into a REDCap database designed for this purpose.

### Variables

Patients switching from MPH (first-line treatment) to LDX were identified and categorized according to whether LDX was second- or third-line treatment. The subpopulation of patients with at least 1 year of observation time after LDX prescription was categorized according to whether they continued LDX treatment for at least 1 year or not.

AEs were classified by System Organ Class and Preferred Term level according to the Medical Dictionary for Regulatory Activities (The International Council for Harmonisation of Technical Requirements for Pharmaceuticals for Human Use, n.d.). The 10 most frequent AEs for LDX, MPH, and ATX were identified. The number of AEs for each patient was categorized into “0–1,” “2–3,” “4–5,” and “>5” AEs. Reasons for discontinuation of medications were categorized into five categories as follows: “lack of efficiency,” “AEs,” “patient/parental request,” “noncompliance,” and “wish for more optimal day coverage.” Reasons were not mutually exclusive.

MPH treatment duration was categorized into “<90 days,” “90–179 days,” “180–365 days,” and “>365 days,” and the number of comorbid conditions was categorized into “0,” “1,” “2,” and “≥3.” The ICD-10 diagnoses were grouped into clinically relevant groups of comorbidity (Table 1). This categorization was performed by experienced clinicians (S.H.R., K.B.N., and N.R.S.). For each dimension of ADHD-rs (inattention and hyperactivity), the sum score and standard deviation were presented.

**Table 1.** Patient Characteristics

		By gender		
	All patients,* N* = 394	Females,* N* = 89	Males,* N* = 305	*p*
Age (years) at time of MPH initiation, mean (SD)	8.3 (2.0)	8.3 (2.0)	8.2 (2.0)	0.692
Diagnosis, *n* (%)				
F90.0, disturbance of activity and attention	331 (84.0)	71 (79.78)	260 (85.25)	**0.048**
F90.1, hyperkinetic conduct disorder	43 (10.9)	9 (10.11)	34 (11.15)	
F98.8c, disturbance of attention without hyperactivity	20 (5.1)	9 (10.11)	11 (3.61)	
ADHD-rs premedication scores, parents, mean (SD)^a^				
Inattention	18.1 (5.1)	18.7 (4.8)	17.9 (5.2)	0.245
Hyperactivity	17.5 (6.2)	17.5 (6.0)	17.5 (6.3)	0.962
ADHD-rs premedication scores, teachers, mean (SD)^b^				
Inattention	19.2 (5.3)	17.4 (5.6)	19.8 (5.1)	**0.002**
Hyperactivity	18.4 (6.7)	15.6 (7.7)	19.1 (6.3)	**0.001**
Psychiatric comorbidity, number, *n* (%)				
0	94 (23.9)	19 (21.4)	75 (24.6)	0.906
1	155 (39.4)	35 (39.3)	120 (39.4)	
2	77 (19.5)	18 (20.2)	59 (19.3)	
≥3	68 (17.3)	17 (19.1)	51 (16.7)	
Comorbidity types (ICD-10 codes), *n* (%)^c^				
Specific developmental disorders (F83, F81, F80, F82)	156 (39.6)	36 (40.4)	120 (39.3)	**0.009**
Pervasive developmental disorders (F84)	62 (15.7)	6 (6.7)	56 (18.4)	
Conduct disorders and mixed conduct and emotional disorders (F91, F92, F98.8, F98.9)	57 (14.5)	13 (14.6)	44 (14.4)	
Cognition disorders and mental retardation (R41.8, F7)	55 (14.0)	12 (13.5)	43 (14.1)	
Affective disorders (F93.8, F32, F41, F93.9, F93.1)	40 (10.2)	11 (12.4)	29 (9.5)	
Stress-related and somatoform disorders (F43, F45)	39 (9.9)	10 (11.2)	29 (9.5)	
Tic disorders (F95)	39 (9.9)	6 (6.8)	33 (10.8)	
Enuresis and encopresis (F98.0, F98.1, R32.9)	30 (7.6)	7 (7.9)	23 (7.5)	
Sleep disorders (G47, F51)	30 (7.6)	11 (12.4)	19 (6.2)	
Obsessive-compulsive disorder (F42)	15 (3.8)	8 (9.0)	7 (2.3)	
Disorders of social functioning (F94)	14 (3.6)	7 (7.9)	7 (2.3)	
Other (F89, F2, F1, F98.5, F98.4, X70.1, R47.8, F99.9, F88)	28 (7.1)	23 (25.8)	5 (1.6)	
MPH treatment duration (first-line treatment), *n* (%)				
<90 Days	87 (22.1)	23 (25.8)	64 (21.0)	0.181
90–179 Days	59 (15.0)	8 (9.0)	51 (16.7)	
180–365 Days	70 (17.8)	13 (14.6)	57 (18.7)	
≥365 Days	178 (45.2)	45 (50.6)	133 (43.6)	
Second-line treatment, *n* (%)				
LDX	255 (64.7)	59 (66.3)	196 (64.3)	0.724
ATX (LDX = third-line treatment)	139 (35.3)	30 (33.7)	109 (35.7)	

Bold indicates significant results (*p* ≤ 0.05).

^a^ 99 Missings.

^b^ 121 Missings.

^c^ Sums up to more than 100% as some patients have more than one comorbid condition.

ADHD-rs, Attention-Deficit/Hyperactive Disorder-Rating Scale; ATX, atomoxetine; ICD-10, International Classification of Diseases, 10th revision; LDX, lisdexamfetamine; MPH, methylphenidate; SD, standard deviation.

### Analysis

Descriptive statistics were used to summarize overall patient and treatment characteristics. Comparison between genders was performed using parametric or nonparametric tests.

For each calendar year, we calculated the percentages of patients who were treated with LDX as second- and third-line treatment. The changes over time in this distribution were tested using chi-squared test. For all types of medications (LDX, MPH, and ATX), the 10 most frequent AEs, the number of AEs, and reason(s) for discontinuation were presented. In patients who discontinued LDX treatment, comparisons were made between LDX and MPH, and between LDX and ATX using parametric or nonparametric tests.

Associations between patient characteristics and the prescription of LDX as third-line rather than second-line treatment were calculated by the use of binomial regression analyses. Unadjusted and adjusted analyses were carried out. In the adjusted analyses, age at time of LDX initiation, gender, and calendar year were included as covariates. Associations were estimated as risk ratios with 95% confidence interval (CI). Similarly, we calculated associations between patient characteristics and LDX treatment lasting a year or longer as opposed to a shorter treatment duration. Finally, the cumulative incidence of switch to LDX from time of MPH initiation was calculated by line of LDX treatment.

A *p* value of ≤0.05 was considered statistically significant. Analyses were conducted using the Stata 15 statistical software program.

### Ethics approval

The project was registered by the Danish Data Protection Agency (Ref. No. 1-16-02-295-20) and conducted in accordance with the Danish Act on Processing of Personal Data. Clearance to analyze the data without obtaining consent from individual patients' parents or guardians was obtained from the Danish health and Medicines Authority (Ref. No. 3-3013-3194/1). According to Danish law, approval by the Danish National Committee on Health Research Ethics was not required as no biomedical intervention was performed [Central Denmark Region (b), n.d.].

## Results

Out of 2473 patients referred to the clinic with a tentative ADHD diagnosis during the study period, 521 (21.1%) patients were prescribed LDX. Patients without a history of medical ADHD treatment before the study period, who switched from MPH (first-line treatment) to LDX (second- or third-line treatment) during the study period, included 394 patients (see Fig. 1 for flowchart).

Patient characteristics are displayed in Table 1. Males and females differed in distribution of ADHD subtype, comorbidity, and on teachers' but not parents' premedication scores on ADHD-rs. More females than males had the inattentive subtype. Pervasive developmental disorders and tic disorders were more common comorbidities in males, whereas affective disorders, stress-related and somatoform disorders, obsessive-compulsive disorder, sleep disorders, and disorders of social functioning were more common comorbidities in females. On teachers' premedication ADHD-rs scores, females had lower mean scores regarding both inattention and hyperactivity compared with males.

LDX was second-line treatment in 255 (64.7%) patients and third-line treatment (after ATX treatment) in 139 (35.5%) patients. In the final 2 years of the study period (2018 and 2019), there was an increase in the proportion of patients prescribed LDX as second-line rather than third-line treatment (71.1% and 86.1%, respectively) (Table 2). The cumulative incidence of LDX prescription (by line of treatment) from time of MPH initiation is shown in Supplementary Figure S1. In patients switching to LDX as second-line treatment, 25% switched to LDX within 114 days and 50% within about 1 year (359 days) from MPH prescription. In patients with LDX as third-line treatment, 25% switched to LDX within about 1 year (361 days) and 50% within 689 days from MPH initiation.

**Table 2.** Lisdexamfetamine Prescriptions According to Calendar Year

	No. of patients prescribed LDX	LDX second-line treatment %	LDX third-line treatment %	*p*
2013^a^	≤5	—	—	**<0.001**
2014	16	56.3	43.7	
2015	43	62.8	37.2	
2016	66	47.0	53.0	
2017	80	52.5	47.5	
2018	106	71.7	28.3	
2019^b^	79	86.1	13.9	

Bold indicates significant results (*p* < 0.05).

LDX may be prescribed later than the year of referral.

^a^ From April 1, 2013.

^b^ Until November 5, 2019.

LDX, lisdexamfetamine.

### Adverse effects

For each type of medication, all registered AEs are presented in Supplementary Appendix Table SA1, whereas the frequency and distribution of the 10 most frequent AEs are reported in Table 3.

**Table 3.** The 10 Most Frequent Adverse Effects, Number of Adverse Effects, and Reasons for Discontinuation of Lisdexamfetamine, Methylphenidate, and Atomoxetine

			Discontinued medical treatment							
	LDX (*N* = 394)		LDX (*N* = 222)		MPH (*N* = 394)			ATX (*N* = 139)		
	No.*^a^*	*n *(%)	No.*^a^*	*n *(%)	No.*^a^*	*n *(%)	*p*-Value (LDX vs. MPH)	No.*^a^*	*n *(%)	*p*-Value (LDX vs. ATX*)*
Type of AE										
Decreased appetite	**1**	246 (62.4)	**1**	140 (63.1)	**1**	330 (83.8)	**<0.001**	**2**	49 (35.3)	**<0.001**
Insomnia	**2**	113 (28.7)	**3**	75 (33.8)	**2**	180 (45.7)	**0.004**	**8**	20 (14.4)	**<0.001**
Irritability/aggression	**3**	103 (26.1)	**2**	86 (38.7)	**3**	154 (39.1)	0.932	**1**	65 (46.8)	0.133
Weight decreased ≥500 g	**4**	83 (21.1)	**4**	61 (27.5)	**4**	128 (32.5)	0.196	**10/11**	14 (10.1)	**<0.001**
Mood swings	**5**	55 (13.9)	**5**	41 (18.5)	**7**	68 (17.3)	0.706	**6**	26 (18.7)	0.955
Depressive symptoms	**6**	48 (12.2)	**6**	39 (17.5)	**5**	86 (21.8)	0.207	**4/5**	28 (20.1)	0.540
Abdominal pain	**7**	48 (12.2)	**8/9**	26 (11.7)	**6**	75 (19.0)	**0.018**	**7**	23 (16.5)	0.192
Rebound effect	**8**	74 (11.9)	**7**	36 (16.2)	**8**	61 (15.5)	0.810		*0*	**<0.001**
Tics	**9**	36 (9.1)	**8/9**	26 (11.7)		*40* (*10.2*)	0.548		*6* (*4.3*)	**0.016**
Headache	**10**	36 (9.1)	**10**	24 (10.8)	**9**	55 (14.0)	0.262	**10/11**	14 (10.1)	0.824
Nausea/vomiting		*33* (*8.4*)		*20* (*9.0*)	**10**	46 (11.7)	0.304	**4/5**	28 (20.1)	**0.002**
Fatigue		*21* (*5.3*)		*15* (*6.8*)		*30* (*7.6*)	0.695	**3**	42 (30.2)	**<0.001**
Anxiety		*15* (*3.8*)		*11* (*5.0*)		*36* (*9.1*)	0.060	**9**	19 (13.7)	**0.004**
Number of AEs										
0–1		135 (34.3)		21 (9.5)		37 (9.3)	0.915		31 (22.3)	**0.002**
2–3		152 (38.6)		91 (41.0)		162 (41.1)			56 (40.3)	
4–5		91 (23.1)		70 (31.5)		132 (33.5)			39 (28.1)	
>5		11 (2.4)		40 (18.0)		63 (16.0)			13 (9.4)	
Reason(s) for discontinuation										
AEs				165 (74.4)		276 (70.0)	0.259		95 (68.3)	0.218
Lack of efficiency				77 (34.7)		205 (52.0)	**<0.001**		101 (72.7)	**<0.001**
Noncompliance				7 (3.2)		19 (4.8)	0.323		7 (5.0)	0.367
Patient/parental request				63 (28.4)		59 (15.0)	**<0.001**		31 (22.3)	0.201
Suboptimal day coverage				21 (9.5)		113 (28.7)	**<0.001**		0	**<0.001**

Italics indicates AEs that are not within the 10 most frequent AEs.

Bold indicates significant results (*p* ≤ 0.05).

^a^ No. = number on the top-10 list of the most frequent AEs.

AE, adverse effect; ATX, atomoxetine; LDX, lisdexamfetamine; MPH, methylphenidate.

The five most frequent AEs for LDX were decreased appetite (62.4%), insomnia (28.7%), irritability/aggression (26.1%), weight decrease (21.1%), and mood swings (13.9%). Overall, the central stimulant treatments (MPH and LDX) had similar AE profiles. However, most AEs were less frequent after switching from MPH to LDX. The difference was statistically significant with regard to decreased appetite, insomnia, and abdominal pain. In general, the AE frequency was higher for LDX and MPH than for ATX. Yet, nausea/vomiting, fatigue, and anxiety were significantly more often reported as an AE of ATX than of the central stimulant treatments. No new AEs or deaths related to LDX were observed, indicating that the safety profile of LDX remained unchanged.

### Reasons for switch to LDX

Reasons for treatment discontinuation according to type of medication are displayed in Table 3. The most frequently stated reason for switching from MPH to either ATX or LDX was “AEs,” followed by “lack of efficiency” and “suboptimal day coverage.” *Post hoc* analysis showed similar percentages in the subpopulation of patients switching from first-line MPH to second-line LDX, with the exception of “lack of efficiency” as a reason for discontinuation for 58.8% of patients in the subpopulation switching to LDX compared with 52.0% of all patients (results not included in Table 3). When the switch was made from second-line ATX to third-line LDX, the most frequently stated reason was “lack of efficiency,” followed by “AEs,” whereas no patients switched from ATX due to “suboptimal day coverage.”

“Patient/parental request” was a reason for discontinuation in 15.0% of patients switching from MPH (15.3% if this switch was made to LDX [results not included in Table 3]) and in 22.3% of patients switching from ATX. “Noncompliance” was a reason for discontinuation in 4.8% of patients switching from MPH (7.1% if this switch was made to LDX [results not included in Table 3]) and in 5.0% of patients switching from ATX to LDX.

### LDX as second- or third-line treatment

Several factors predicted LDX as third-line treatment rather than second-line treatment. These included being diagnosed with F98.8c (disturbance of attention without hyperactivity) rather than F90.0 (disturbance of activity and attention), an increased number of psychiatric comorbidities, higher parental ADHD-rs scores, increased number of MPH AEs, and if “AEs” were a reason for discontinuation of MPH (Table 4). In contrast, the likelihood of LDX as third-line decreased if “lack of efficiency” was a reason for MPH discontinuation.

**Table 4.** Patient Characteristics in Relation to Whether Lisdexamfetamine Was Prescribed as Third-Line Treatment Rather than Second-Line Treatment

	RR (95% CI)	Adjusted RR*^a^* (95% CI)
Gender, *n* (%)		
Female	1	1
Male	1.01 (0.76–1.47)	1.06 (0.77–1.47)
Age at time of LDX prescription	**1.08 (1.01–1.15)**	1.07 (1.00–1.14)
Diagnosis		
F90.0, disturbance of activity and attention	1	1
F90.1, hyperkinetic conduct disorder	1.33 (0.91–1.92)	1.38 (0.96–1.98)
F98.8c, disturbance of attention without hyperactivity	1.50 (0.94–2.29)	**1.51 (1.04–2.17)**
Psychiatric comorbidity, Number		
0	1	1
1	1.37 (0.90–2.08)	1.30 (0.86–1.98)
2	**1.75 (1.13–2.71)**	**1.65 (1.07–2.55)**
≥3	**1.86 (1.20–2.89)**	**1.87 (1.21–2.89)**
ADHD-rs premedication scores, parents^b^		
Inattention	**1.04 (1.01–1.07)**	**1.05 (1.02–1.08)**
Hyperactivity	**1.04 (1.02–1.07)**	**1.05 (1.02–1.07)**
ADHD-rs premedication scores, teachers^c^		
Inattention	0.98 (0.95–1.00)	0.98 (0.95–1.00)
Hyperactivity	0.98 (096–1.00)	0.98 (0.96–1.00)
Reason(s) for discontinuation of MPH		
AEs	**1.62 (1.15–2.30)**	**1.63 (1.16–2.31)**
Lack of efficiency	**0.62 (0.47–0.81)**	**0.64 (0.49–0.84)**
Noncompliance	0.29 (0.08–1.07)	0.27 (0.07–1.01)
Patient/parental request	0.95 (0.65–1.04)	0.95 (0.65–1.38)
Suboptimal day coverage	1.23 (0.93–1.62)	1.15 (0.87–1.53)
MPH AEs, No.		
0–1	1	1
2–3	1.73 (0.86–3.49)	1.79 (0.89–3.58)
4–5	1.88 (0.93–3.81)	1.93 (0.96–3.89)
>5	**2.68 (1.32–5.46)**	**2.60 (1.28–5.27)**

Bold indicates significant results (*p* ≤ 0.05).

^a^ Adjusted for gender, age at time of LDX prescription, and calendar year.

^b^ 99 Missings.

^c^ 121 Missings.

ADHD-rs, Attention Deficit/Hyperactive Disorder-Rating Scale; AE, adverse effect; CI, confidence interval; LDX, lisdexamfetamine; MPH, methylphenidate; RR, risk ratio.

All recorded types of comorbidity (as presented in Table 1), with the exception of “enuresis and encopresis,” were more common in patients prescribed LDX as third-line treatment compared with second-line treatment. Yet, this difference was significant for anxiety only; in patients prescribed LDX as third-line treatment, 17.2% (95% CI: 11.4%–24.6%) were diagnosed with anxiety, whereas 6.2% (95% CI: 3.6%–9.9%) of patients with LDX as second-line treatment had this diagnosis (results not included in Table 4).

### Discontinuation of LDX treatment

At the end of the study period, LDX was discontinued in 58.0% of patients with LDX as second-line treatment and in 53.2% of patients with LDX as third-line treatment (Fig. 1).

The most frequent reasons for LDX discontinuation were “AEs,” followed by “lack of efficiency” and “patient/parental request” (Table 3). Yet, “lack of efficiency” was a less frequent reason for discontinuation of LDX compared with both MPH and ATX. “Suboptimal day coverage” as a reason for discontinuation of treatment was less frequent for LDX compared with MPH, but more frequent than for ATX.

In the subpopulation of 298 patients with at least 1 year of observation time from LDX prescription, 41.3% continued LDX treatment for 1 year or longer.

LDX treatment for more than 1 year was less likely if patients had one comorbidity rather than none and if “AEs” were a reason for MPH discontinuation (Table 5). Other reasons for MPH discontinuation tended to increase the likelihood of LDX treatment for more than 1 year; however, these results were not statistically significant.

**Table 5.** Patient and Treatment Characteristics in Relation to Whether Patients Were Treated with Lisdexamfetamine for ≥1 Year

	RR (95% CI)	Adjusted RR*^a^* (95% CI)
Second-line treatment, *n* (%)		
LDX	1	1
ATX (LDX = third-line treatment)	0.91 (0.67–1.21)	0.99 (0.74–1.32)
Gender, *n* (%)		
Female	1	1
Male	0.97 (0.71–1.33)	1.06 (0.80–1.41)
Age at time of LDX prescription	0.93 (0.86–1.00)	0.98 (0.90–1.06)
Diagnosis		
F90.0, disturbance of activity and attention	1	1
F90.1, hyperkinetic conduct disorder	1.34 (0.94–1.90)	1.20 (0.92–1.57)
F98.8c, disturbance of attention without hyperactivity	1.12 (0.53–2.37)	1.41 (0.77–2.60)
Psychiatric comorbidity, Number		
0	1	1
1	**0.63 (0.45–0.89)**	**0.71 (0.51–0.97)**
2	0.79 (0.54–1.16)	0.91 (0.65–1.26)
≥3	0.80 (0.55–1.16)	0.91 (0.67–1.24)
ADHD-rs premedication scores, parents^b^		
Inattention	0.98 (0.95–1.01)	0.98 (0.95–1.02)
Hyperactivity	0.99 (0.96–1.02)	0.99 (0.96–1.02)
ADHD-rs premedication scores, teachers^c^		
Inattention	1.03 (0.99–1.08)	1.04 (1.00–1.08)
Hyperactivity	1.00 (0.98–1.04)	1.00 (0.97–1.04)
Reason(s) for discontinuation of MPH		
AEs	**0.69 (0.53–0.91)**	**0.51 (0.30–0.87)**
Lack of efficiency	1.25 (0.95–1.65)	1.42 (0.87–2.31)
Noncompliance	1.41 (0.88–2.27)	2.09 (0.66–6.59)
Patient/parental request	1.18 (0.83–1.68)	1.39 (0.71–2.75)
Suboptimal day coverage	1.19 (0.89–1.59)	1.47 (0.85–2.53)
MPH AEs, Number		
0–1	1	1
2–3	1.10 (0.68–1.77)	1.21 (0.49–2.97)
4–5	0.94 (0.57–1.54)	0.93 (0.37–2.34)
>5	**0.48 (0.28–0.68)**	0.45 (0.15–1.35)

*N* = 298 patients with ≥1 year of observation from the time of LDX prescription. Bold indicates significant results (*p* ≤ 0.05).

^a^ Adjusted for gender, age at time of LDX prescription, and calendar year.

^b^ 73 Missings.

^c^ 96 Missings.

ADHD-rs, Attention Deficit/Hyperactive Disorder-Rating Scale; AE, adverse effect; ATX, atomoxetine; CI, confidence interval; LDX, lisdexamfetamine; MPH, methylphenidate; RR, risk ratio.

## Discussion

To the best of our knowledge, this study is the first to provide information on frequency as well as reasons for switch to LDX treatment in school-aged children (7–13 years) with ADHD. The study period started at the time of LDX marketing in Denmark and provides insights into the course of LDX prescription patterns in a specialized clinical setting.

During the study period, one in five children referred with a tentative ADHD diagnosis were prescribed LDX as either second- or third-line treatment. In line with this, a German study found that LDX prescriptions accounted for 23% of all prescriptions for ADHD in children/adolescents in 2018 (Grimmsmann and Himmel, 2021). Similarly, there has been a rise in the use of LDX in the United States following its approval by the FDA for treatment of ADHD in children aged 6–12 years in 2007 and for adolescents aged 13–17 years in 2010 (Department of Health and Human Services et al., 2020; Piper et al., 2018).

Our finding that LDX was more frequently prescribed as second-line rather than third-line treatment by the end of the study period (2018 and 2019), in contrast to a more even distribution in previous years, aligns with the update of the NICE guideline in 2018 (National Institute for Health and Care Excellence, 2018). Before this update, ATX was recommended as second-line treatment, while the updated guideline recommends LDX as second-line treatment. Prescribers gaining clinical experience with LDX and the slower onset of effects with ATX may have contributed to the more frequent prescription of LDX as second-line treatment in the later years of the study. Gain of clinical experience with LDX could also affect how soon a switch to LDX from MPH or ATX is made.

As the study period started at the time of LDX marketing in Denmark, this should be held in mind in the interpretation of Supplementary Figure S1 showing the cumulative incidence of switch to LDX from time of MPH initiation.

### Reasons for switch to and from LDX

Our finding that the most frequent reasons for discontinuation of ADHD medication were “AEs” and “lack of efficiency” is in line with others studies (Prasad and Steer, 2008; Toomey et al., 2012). We found “lack of efficiency” to be a more frequent reason for ATX discontinuation (72.7%) than it was for MPH (52.0%) and LDX (34.7%) discontinuation. This is in concordance with current evidence suggesting that LDX and MPH are more effective than ATX (Dittmann et al., 2014; Dittmann et al., 2013; Faraone et al., 2006; Nagy et al., 2016; Soutullo et al., 2013). Some studies suggest that LDX is superior to both ATX and MPH (Soutullo et al., 2013). These differences in treatment response may explain our finding that LDX was more often chosen as second-line treatment if “lack of efficiency” was a reason for MPH discontinuation.

Studies have shown that the response to ADHD medication varies among individual patients, and that a nonresponse to one type of psychostimulant does not predict a nonresponse to a second (Dittmann et al., 2014; Hodgkins et al., 2012).

Our finding that “wish for more optimal day coverage” was a reason for switching from MPH to LDX (28%) but not for switching from ATX to LDX was expected, as single-dose ATX can provide a 24-hour effect (Clemow and Bushe, 2015). A Danish study on children switching from MPH to ATX found that a wish for optimal day coverage was a reason for making this switch in 23.6% of the children (Warrer et al., 2016). Our finding that a higher parental ADHD-rs score was associated with increased likelihood of ATX as second-line treatment could also reflect a wish for full day coverage among some of these patients.

Potential substance abuse has previously been reported as a reason for switch (Prasad and Steer, 2008). This was not stated as a reason for switch in our study, which may be because of the low age of our sample.

### Adverse effects

Our finding that the most frequent AEs of LDX were decreased appetite (62.4%), insomnia (28.7%), irritability/aggression (26.1%), weight decrease (21.1%), and mood swings (13.9%) was expected and consistent with product labeling (Medicine and Health care Products Regulatory) and other study findings (Findling et al., 2013; Findling et al., 2008; Ichikawa et al., 2020). However, one study reported lower frequency of some AEs compared with our study, with, for example, decreased appetite in 25.8% and insomnia in 11.7% of children treated with LDX, which could be due to a shorter follow-up period (Dittmann et al., 2013).

Long-term studies found that most AEs occur during the first weeks of treatment and suggest that patients become accustomed to LDX with continued use (Ichikawa et al., 2020). Some symptoms (e.g., insomnia irritability/aggression and mood swings) registered as AEs might be due to other factors than medication, for example, comorbid psychiatric conditions or suboptimal treatment of ADHD. Yet, the high prevalence of such symptoms in our study underlines the importance of assessing these symptoms when evaluating and making decisions about medical treatment.

The similarities in LDX and MPH profiles of AEs were expected. It was interesting, however, that most AEs were less frequent after switching from MPH to LDX. Thus, some patients experienced specific AEs to MPH and not to LDX. It should be kept in mind, though, that all patients in our study discontinued MPH treatment. Therefore, the frequency of MPH AEs is likely to be higher in our population compared with a population that includes children who continue MPH treatment.

LDX discontinuation within 1 year of treatment was more likely if “AEs” were a reason for MPH discontinuation, which may suggest that significant MPH AEs may be associated with less favorable treatment outcomes of LDX. If this is an observation among the clinicians, it could explain the increased probability of LDX as third-line rather than second-line treatment with increasing number of MPH AEs and when “AEs” were a reason for MPH discontinuation. In contrast, other studies have suggested that the outcome of treatment with one ADHD medication does not predict the success of treatment with another (Hodgkins et al., 2012).

In general, the AE frequency was lower for ATX than for LDX and MPH. Other studies have shown similar tolerability for ATX and stimulants (Gibson et al., 2006). In line with other studies, no severe AEs were observed in this study (Biederman et al., 2007; Coghill et al., 2014; European Medicines Agency, n.d.; Findling et al., 2013; Ichikawa et al., 2020; Newcorn et al., 2017; van Stralen et al., 2023).

### LDX as second- versus third-line

The likelihood of LDX as third-line treatment increased with the number of psychiatric comorbidities. An evidence review in relation to the update of NICE guidelines in 2018 reported that historically, clinicians have been hesitant to use stimulant medication in individuals with psychiatric comorbidities (e.g., anxiety disorder, tic disorder, or autism spectrum disorder) for fear of worsening these conditions, but found no evidence supporting this [National Guideline Centre (UK), 2018]. However, a systemic review from 2022 concludes that ATX demonstrates significant efficacy in improving anxiety symptoms in children and adolescents with ADHD (Khoodoruth et al., 2022).

### LDX discontinuation

Our finding that 41.3% of children continued LDX treatment for at least 1 year supports previous findings that LDX may provide an alternative treatment option for some children with inadequate response to previous ADHD medication (Dittmann et al., 2014; Dittmann et al., 2013; Frampton, 2018; Maneeton et al., 2015; Nagy et al., 2016).

“Patient/parental request” was a reason for LDX discontinuation in about one-third of the patients. We have no information on whether they switched back to MPH or ATX, paused treatment to resume LDX treatment at a later time, or discontinued medical treatment for ADHD all together. A study of adolescents with ADHD found that the number of patients treated with any type of ADHD medication decreased with age (Farahbakhshian et al., 2021). Some patients may discontinue LDX treatment due to perceived resolution of ADHD symptoms (Sibley et al., 2022).

A study on LDX utilization patterns in children, adolescents, and adults in eight European countries during a study period of about 5 years from the launch of LDX reported that between 22.8% and 70.6% of patients discontinued LDX without switching to another type of ADHD medication, and between 10.7% and 33.8% of patients switched from LDX to other types of ADHD medications (Siffel et al., 2020).

### Strengths and limitations

A strength of this study is the use of data from patient records throughout a predetermined study period. The study findings reflect “real-world” clinical practice at a Danish child and adolescent psychiatric outpatient clinic. The findings are particularly generalizable to countries where LDX is approved as second-line treatment when response to MPH is considered clinically inadequate. Throughout the interpretation of the results, it should be kept in mind that all patients discontinued MPH treatment. In clinical practice, different formulations of MPH (e.g., immediate or modified release) are often tried before switching. Switch of medication can be assumed to relate to suboptimal outcome of the index treatment (Ben Amor et al., 2014; Childress and Sallee, 2014; Gajria et al., 2014). This could indicate higher levels of psychiatric complexity and increased risk of suboptimal treatment outcome of another type of medication.

In adolescents with ADHD, the presence of psychiatric comorbidities has been found to be associated with increased likelihood of switch of treatment (Farahbakhshian et al., 2021). Thus, the efficiency and AE profiles reported in this study could be expected to be less favorable than in studies that include children who do not switch from first-line treatment.

The extraction of data from patient records included valuable information on, for example, AEs and reasons for treatment discontinuation. However, data validity depended on the quality of information retrieved and registered by the clinicians. Thus, the possibility of missing information cannot be ruled out. However, the collection of information during follow-up visits was systemized and included ADHD-rs scores from teachers and parents and information on AEs. Information on severity and duration of AEs was not retrieved, and some symptoms registered as AEs may relate to other factors than medication. However, there is no reason to believe that there are systematic differences in the registration of AEs according to type of medication.

We did not collect information about the different formulations of MPH, nor did we collect information about the dosing regimen or type of switching (immediate switching, cross tapering, or gap switching) or combination treatment.

Due to the retrospective observational methodology of the study, causal relationships cannot be inferred.

## Conclusions

In conclusion, about one in five patients switched from first-line MPH to LDX. At the end of the study period, the majority were prescribed LDX as second-line rather than third-line treatment. Regardless of medication type, “AEs” and “lack of efficiency” were the most frequent reasons for treatment discontinuation. Most frequent AEs of LDX were decreased appetite, insomnia, irritability/aggression, weight decrease, and mood swings. MPH and LDX had similar AE profiles, yet most AEs were less frequent after switching to LDX.

LDX was more often chosen as third-line rather than second-line treatment in patients with more psychiatric comorbidities, higher ADHD symptom severity as assessed by parents, and if “AEs” were a reason for MPH discontinuation. About four out of ten patients continued treatment for more than 1 year after switching to LDX, yet LDX continuation was less likely if “AEs” were a reason for MPH discontinuation.

## Clinical Significance

Our findings may support prescribers in the clinical decision-making regarding prescription of LDX for ADHD in school-aged children. Nonetheless, further studies are needed to provide insights for making informed decisions about medication selection, including knowledge to help prescribers more effectively identify patients who might or might not benefit from a switch to LDX. Future studies should ideally include a nonswitcher comparator group.

## Supplementary Material

Supplemental data

Supplemental data

## Supplementary Material

Supplementary Figure S1

Supplementary Appendix Table SA1
