# Supplementary figures and images for: Switch to Lisdexamfetamine in the Treatment of Attention-Deficit Disorder at a Psychiatric Outpatient Clinic for School-Aged Children: A Danish Cohort Study
Source: J Child Adolesc Psychopharmacol. 2024 Apr 11;34(3):137–47. doi: 10.1089/cap.2023.0077 (PMC11040185; doi:10.1089/cap.2023.0077)

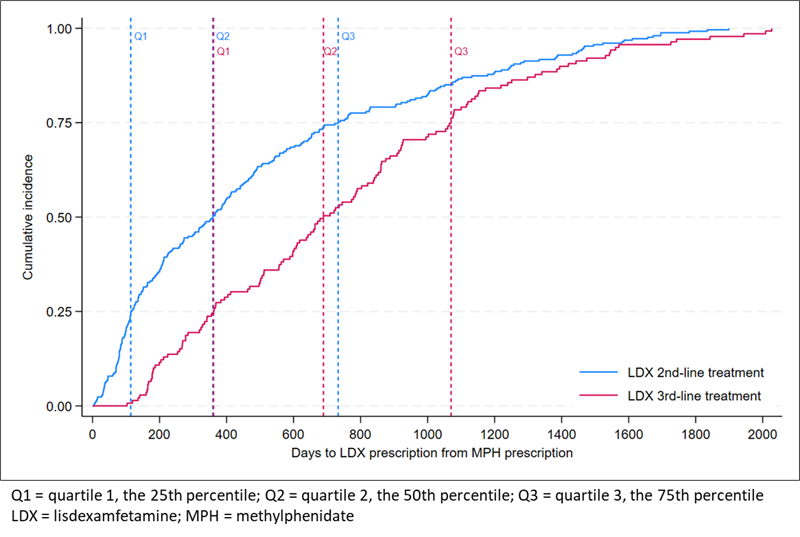

Supplement: Supplemental data [file Suppl_FigS1.png]
