# Supplementary material for: Switch to Lisdexamfetamine in the Treatment of Attention-Deficit Disorder at a Psychiatric Outpatient Clinic for School-Aged Children: A Danish Cohort Study
Source: J Child Adolesc Psychopharmacol. 2024 Apr 11;34(3):137–47. doi: 10.1089/cap.2023.0077 (PMC11040185; doi:10.1089/cap.2023.0077)
Supplement: Supplemental data [file Suppl_AppTableSA1.docx]

**Supplementary appendix table.** Frequency of adverse effects according to drug type.

| **System organ class (SOC)**  Preferred term (PT) | |  | **Discontinued medical treatment** | | |
| --- | --- | --- | --- | --- | --- |
|  |  | LDX  (N=394) | LDX  (N=222) | MPH  (N=394) | ATX  (N=139) |
|  |  | n (%) | n (%) | n (%) | n (%) |
| **Metabolism and nutrition disorders** | |  |  |  |  |
|  | Decreased appetite | 246 (62.4) | 140 (63.1) | 330 (83.8) | 49 (35.3) |
|  | Growth retardation | 11 (2.8) | 10 (4.5) | 10 (2.5) | <5 |
| Investigations | |  |  |  |  |
|  | Weight decreased (≥500 g) | 83 (21.1) | 61 (27.5) | 128 (32.5) | 14 (10.1) |
|  | Body temperature increased | 0 | 0 | 0 | 0 |
|  | Heart rate increased | 9 (2.3) | 6 (2.7) | 10 (2.5) | 10 (7.2) |
|  | Blood pressure increased | <5 | <5 | 6 (1.5) | 6 (4.3) |
|  | Electrocardiogram QT prolonged | 0 | 0 | 0 | 0 |
| Gastrointestinal disorders | |  |  |  |  |
|  | Nausea/vomiting | 33 (8.4) | 20 (9.0) | 46 (11.7) | 28 (20.1) |
|  | Abdominal pain | 48 (12.2) | 26 (11.7) | 75 (19.0) | 23 (16.5) |
|  | Defaecation disorder | <5 | 0 | 5 (1.3) | <5 |
|  | Dry mouth | <5 | <5 | <5 | <5 |
|  | Salivary hypersecretion | <5 | <5 | 0 | 0 |
| **General disorders and administration site conditions** | |  |  |  |  |
|  | Fatigue | 21 (5.3) | 15 (6.8) | 30 (7.6) | 42 (30.2) |
|  | Rebound effect | 47 (11.9) | 36 (16.2) | 61 (15.5) | 0 |
|  | Chest pain | 8 (2.0) | 5 (2.3) | 5 (1.3) | <5 |
|  | Sudden death | 0 | 0 | 0 | 0 |
| Musculoskeletal and connective tissue disorders | |  |  |  |  |
|  | Myalgia/arthralgia | <5 | 0 | <5 | <5 |
| Nervous system disorders | |  |  |  |  |
|  | Headache | 36 (9.1) | 24 (10.8) | 55 (14.0) | 14 (10.1) |
|  | Dizziness | 11 (2.8) | 7 (3.2) | 9 (2.3) | <5 |
|  | Insomnia | 113 (28.7) | 75 (33.8) | 180 (25.7) | 20 (14.4) |
|  | Tics | 36 (9.1) | 26 (11.7) | 40 (10.2) | 6 (4.3) |
|  | Syncope | 0 | 0 | 0 | 0 |
|  | Seizure | <5 | <5 | <5 | <5 |
|  | Vision blurred | 0 | 0 | <5 | 0 |
|  | Muscle rigidity | 0 | 0 | 0 | 0 |
| Renal and urinary disorders | |  |  |  |  |
|  | Lower urinary tract symptoms | <5 | <5 | <5 | <5 |
| Reproductive system and breast disorders | |  |  |  |  |
|  | Breast discomfort | <5 | 0 | 0 | 0 |
|  | Menstrual disorder | 0 | 0 | 0 | 0 |
| Skin and subcutaneous tissue disorders | |  |  |  |  |
|  | Rash | 5 (1.3) | 5 (2.3) | 12 (3.0) | <5 |
| Vascular disorders | |  |  |  |  |
|  | Cerebrovascular accident | 0 | 0 | 0 | 0 |
|  | Myocardial ischaemia | 0 | 0 | 0 | 0 |
|  | Raynaud's phenomenon | 0 | 0 | 0 | 0 |
| **Cardiac disorders** | |  |  |  |  |
|  | Palpitations | 8 (2.0) | 7 (3.2) | 11 (2.8) | 5 (3.6) |
|  | Cardiomyopathy | 0 | 0 | 0 | 0 |
| Psychiatric disorders | |  |  |  |  |
|  | Irritability/aggression | 103 (26.1) | 86 (38.7) | 154 (39.1) | 65 (46.8) |
|  | Psychotic disorder | <5 | <5 | 5 (1.3) | <5 |
|  | Completed suicide | 0 | 0 | 0 | 0 |
|  | Mood alterations with depressive symptoms | 48 (12.3) | 39 (17.6) | 86 (21.8) | 28 (20.1) |
|  | Anxiety | 15 (3.8) | 11 (5.0) | 36 (9.1) | 19 (13.7) |
|  | Suicidal ideation | 9 (2.3) | 9 (4.1) | 12 (3.0) | 9 (6.5) |
|  | Mood swings | 55 (14.0) | 41 (18.5) | 68 (17.3) | 26 (18.8) |
|  | Attention deficit hyperactivity disorder | <5 | <5 | 6 (1.5) | 7 (5.0) |
|  | Thinking abnormal | 26 (6.6) | 20 (9.1) | 25 (6.3) | 11 (7.9) |
| Other | | 21 (5.3) | 16 (7.2) | 34 (8.6) | 15 (10.8) |

LDX, lisdexamfetamine; MHP, methylphenidate; ATX, atomoxetine
